# A Meta-Analysis of Randomized Controlled Trials of Yiqi Yangyin Huoxue Method in Treating Diabetic Nephropathy

**DOI:** 10.1155/2016/3257603

**Published:** 2016-05-30

**Authors:** Jiao Ying Ou, Di Huang, Yan Sheng Wu, Lin Xu, Fei He, Hui Ling Wang, Li Qiang Shi, Qiang Wan, Li Qun He, Jian Dong Gao

**Affiliations:** ^1^Department of Nephrology, Shuguang Hospital, Shanghai University of Traditional Chinese Medicine, No. 528 Zhang Heng Road, Shanghai 201203, China; ^2^TCM Institute of Kidney Disease, Shanghai University of Traditional Chinese Medicine, No. 528 Zhang Heng Road, Shanghai 201203, China; ^3^Shanghai Key Laboratory of Traditional Chinese Clinical Medicine, No. 528 Zhang Heng Road, Shanghai 201203, China; ^4^Department of Internal Medicine, Shanghai TCM-Integrated Hospital of Shanghai University of Traditional Chinese Medicine, No. 184 Baoding Road, Shanghai 200082, China

## Abstract

*Objective*. The purpose of this systematic review is to evaluate the evidence of Yiqi Yangyin Huoxue Method for diabetic nephropathy.* Methods*. 11 electronic databases, through September 2015, were searched to identify randomized controlled trials of Yiqi Yangyin Huoxue Method for diabetic nephropathy. The quality of the included trials was assessed using the Jadad scale.* Results*. 26 randomized controlled trials were included in our review. Of all the included trials, most of them were considered as high quality. The aggregated results suggested that Yiqi Yangyin Huoxue Method is beneficial to diabetic nephropathy in bringing down the microalbuminuria (SMD = −0.98, 95% CI −1.22 to −0.74), serum creatinine (SMD = −0.56, 95% CI −0.93 to −0.20), beta-2 microglobulin (MD = 0.06, 95% CI 0.01 to 0.12), fasting plasma glucose (MD = −0.35, 95% CI −0.62 to −0.08), and 2-hour postprandial blood glucose (MD = 1.13, 95% CI 0.07 to 2.20), but not in decreasing blood urea nitrogen (SMD = −0.72, 95% CI −1.47 to 0.02) or 2-hour postprandial blood glucose (SMD = −0.48, 95% CI −1.01 to 0.04).* Conclusions*. Yiqi Yangyin Huoxue Method should be a valid complementary and alternative therapy in the management of diabetic nephropathy, especially in improving UAER, serum creatinine, fasting blood glucose, and beta-2 microglobulin. However, more studies with long follow-up are warrant to confirm the current findings.

## 1. Introduction

Diabetic nephropathy (DN) is one of the common complications of diabetes. Due to the increase in the global prevalence of diabetes, the incidence of diabetic nephropathy is rising year by year, the data has shown that there are 30–40% type 1 diabetes mellitus (T1DM) and type 2 diabetes mellitus (T2DM) for the development of DN [1]. In Western countries the ratio is higher, about 37%–40% of the patients with DN ESRD, which is currently the primary cause of end-stage renal disease [2]. In China, about 30% of type 1 diabetes and 20% of type 2 diabetes developed into DN, of which about 53% of the patients died of renal failure caused by diabetic nephropathy [3]. For hundreds of years, Chinese clinicians have accumulated rich clinical experience in the treatment of chronic kidney disease (CKD) and DN.

It has been recorded that Chinese medicine had been used to treat CKD and DN for at least two thousand years. Nowadays, Chinese herbal medicine is still widely used in the treatment of chronic kidney disease and DN. Herbs that act to tonify qi and yin and invigorate blood (Yiqi Yangyin Huoxue) were beneficial for DN in many clinical trials. The studies of Yiqi Yangyin Huoxue Method for diabetic nephropathy have been conducted; however, owing to the low quality of the reported trials, the effect of Yiqi Yangyin Huoxue Method for diabetic nephropathy remains less defined. Therefore, the purpose of this review is to evaluate the evidence of Yiqi Yangyin Huoxue Method for diabetic nephropathy by investigating related trials. To our knowledge, this is a comprehensive review summarizing the efficacy of Yiqi Yangyin Huoxue Method for diabetic nephropathy focusing on the urinary albumin excretion rate (UAER), serum creatinine (SCr), urine beta-2 microglobulin, fasting plasma glucose (FBG), and so forth. Based on our findings, scientific evaluation on the efficacy of Yiqi Yangyin Huoxue Method for diabetic nephropathy will be offered.

## 2. Methods

### 2.1. Search Strategy

The following electronic databases through September 2015 were searched: China Knowledge Resource Integrated (CNKI), Wanfang Data, VIP, Social Science Journal, Sinomed, PubMed, EMBASE, Cochrane Library, Sciverse science direc, Springer, and OVID. The following key words were used in combination, Yiqi Yangyin Huoxue, Supplementing qi, nourishing yin, activating blood circulation, diabetic nephropathy, diabetes, kidney disease, renal disease, and random. The reference lists of selected reviews were also screened. In order to identify unpublished studies, dissertations and trial registrations were searched. And we contacted experts in the field.

### 2.2. Study Selection

#### 2.2.1. Inclusion Criteria

The studies that met the following criteria were included: (1) study design included randomized controlled trials (RCTs) or quasirandomized controlled trials; (2) the target population was patients diagnosed with diabetic nephropathy (Mogensen I–IV) [4]; (3) the main intervention should be Yiqi Yangyin Huoxue herbs plus routine treatment compared with control group with only routine treatment (diabetic diet plus exercise plus hypoglycemic drugs or insulin); (4) the outcomes included the microalbuminuria excretion, serum creatinine, urine beta-2 microglobulin, and fasting plasma glucose; (5) the study was available in either English or Chinese.

#### 2.2.2. Exclusion Criteria

The trials that met the following criteria were excluded: (1) Yiqi Yangyin Huoxue herbs were used as an adjuvant treatment; (2) the target population was incongruent with diagnostic criteria of DN; (3) the main intervention was mixed with too many measures; (4) the study was not allocated with appropriate comparator or without randomization; (5) the studies with data unavailable or duplicate publication were excluded.

#### 2.2.3. Intervention

The treatment group and control group were all given the routine treatment for diabetic nephropathy (diabetic diet plus exercise plus hypoglycemic drugs or insulin), while the treatment was intervened by Yiqi Yangyin Huoxue herbs on the basis of routine treatment.

### 2.3. Literature Screening

Literature screening was performed by two authors (Di Huang and Jian Dong Gao) independently. They screened literature on the inclusion criteria. All identified abstracts and full texts of potentially relevant abstracts were screened independently by two authors. The corresponding author was contacted when relevant information was not reported. And disagreements were resolved by consensus among authors.

### 2.4. Methodological Quality Assessment

The quality of the eligible studies was assessed by Jadad scale [5]. It assesses the quality of published clinical trials based Methods relevant to random assignment, double blinding, and the flow of patients. There are 11 items. The range of possible scores is 0 (bad) to 5 (good) (the trial that attracts 1-2 scores was considered as low quality, while that with 3–5 means high quality) [6, 7]: (1) was the Method used to generate the sequence of randomization described? (2) Was the Method used to generate the sequence of randomization described as computer-generated? (+2 Points); (3) the study was described as randomized, but the Method used to generate the sequence of randomization was not described (+1 Point); (4) the study was described as quasirandomized (+0 Points); (5) was the study described as double blind? (+1 Point); (6) was the Method of double blinding described and appropriate (identical placebo, active placebo, dummy, etc.)? (+2 Points); (7) the Method of double blinding was described only (+1 Point); (8) the Method of double blinding was described, but inappropriate (+0 Points); (9) was there a description of withdrawals and dropouts? (10) the withdrawals and dropouts were described in detail (+1 Point); and (11) the withdrawals and dropouts were not mentioned (+0 Points). The corresponding author was contacted when relevant information was not reported.

### 2.5. Data Abstraction

Three reviewers independently extracted data onto predefined criteria in Table 1. The information extracted from the participants was gender, age, nation, diagnosis Method, and course of disease. The outcome measures extracted from the trial were microalbuminuria, serum creatinine, urine beta-2 microglobulin, fasting plasma glucose, and so forth.

### 2.6. Data Synthesis and Analysis

We employed the Cochrane Collaboration software (Review Manager Version 5.3 for Windows; Copenhagen: The Nordic Cochrane Centre) for meta-analysis. The heterogeneity was assessed with chi-square test. If the heterogeneity test results were *p* > 0.05, then there is homogeneity in multiple independent trials and fixed effect model was adopted. If heterogeneity test results were *p* ≤ 0.05, then there is heterogeneity in multiple independent studies and the random effect model was used. The mean differences and 95% confidence interval were adopted as the effect of expression. The effect size was tested with hypothesis test—*z* test. If *p* ≤ 0.05, there is a statistical significance in the research of multiple combined statistics; if *p* > 0.05, there is no statistical significance in the merged statistic. When the experimental effect index value is equal to 0, the MD experiment effect is invalid, which means its 95% CI contains 0 and it is equivalent to *p* > 0.05; namely, there is no statistical significance, if the upper and lower limits do not contain 0 (were greater than 0 or less than 0), which means being equivalent to *p* < 0.05 and it is statistically significant. The confidence of estimates of effect was assessed based on GRADE Working Group grades of evidence, high quality: further research is very unlikely to change our confidence in the estimate of effect; moderate quality: further research is likely to have an important impact on our confidence in the estimate of effect and may change the estimate; low quality: further research is very likely to have an important impact on our confidence in the estimate of effect and is likely to change the estimate; and very low quality: we are very uncertain about the estimate.

## 3. Results

### 3.1. Literature Search

**Figure 1:**
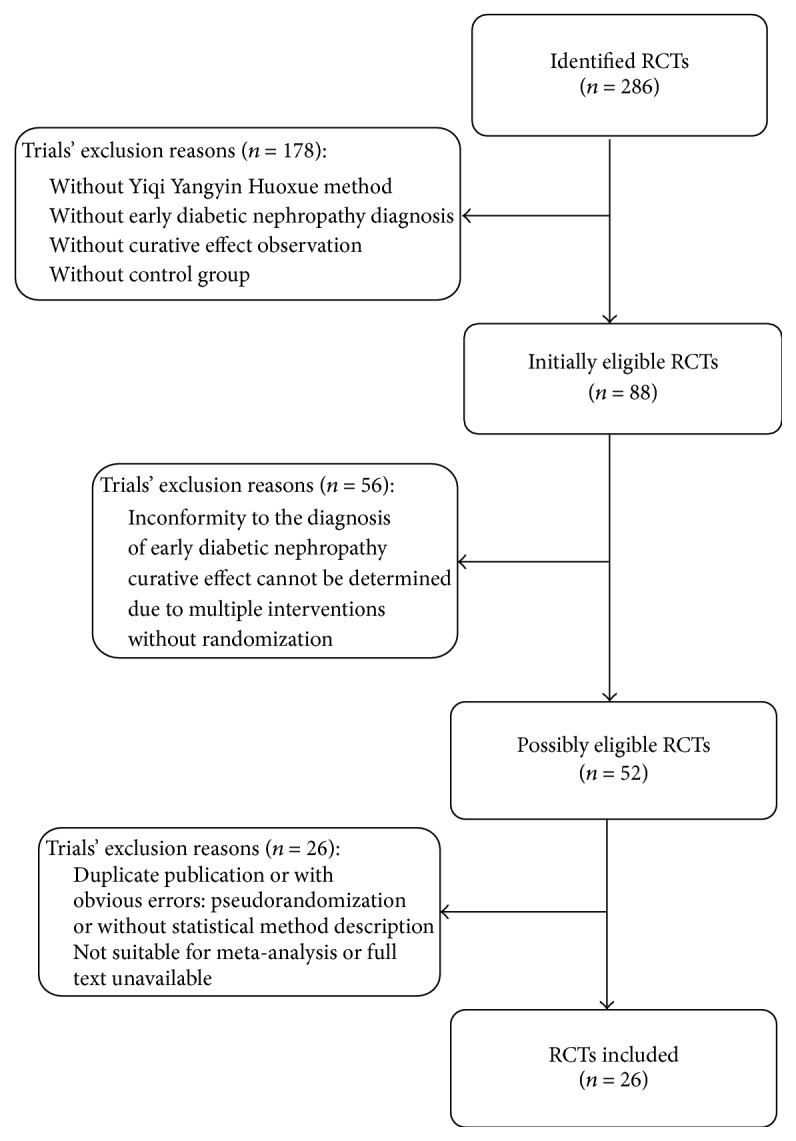
Flow diagram.

286 records were identified from 11 English and Chinese databases (Figure 1). After removing duplicates, 108 potentially relevant abstracts were initially screened, and 178 were excluded for failing to meet the inclusion criteria. We retrieved and reviewed 52 full-text articles. 26 [8–33] studies were excluded due to nonrandomized, duplicate publications, suspicion of counterfeit, and failure to get available data. 26 RCTs of them were eligible [34–59]. No dissertations and trial registrations were obtained. The characteristic of selected studies was shown in Table 1.

### 3.2. Study Characteristics

17 out of the 26 RCTs were high quality trials, one for 5 points, eight for 4 points, and eight for 3 points and the rest of the 9 were relatively low, only 2 points. Randomization was conducted in all articles, of which there are 2 with allocation concealment; 7 mentioned the blinding and the withdrawals and dropouts or resign were mentioned in 4 trials. We were not able to perform a sensitivity analysis on the quality of the trials due to the shortage of single-end report (Table 2).

### 3.3. Quantitative Data Synthesis

#### 3.3.1. Microalbuminuria (UAER)

According to the urine microprotein changes in forest plot, the effect size of the 22 selected trials [35–37, 39–44, 46–52, 54–59] was SMD = −0.98 and 95% CI −1.22 to −0.74; test of the effect size was *z* = 8.02 and *p* < 0.00001; and the heterogeneity analysis (*p* < 0.05) suggests that there is heterogeneity in 22 articles; therefore random effect model was used. The aggregated results of 21 RCTs [35, 36, 39–44, 46–52, 54–59] suggested that Yiqi Yangyin Huoxue Method showed favorable effects for decreasing microalbuminuria of diabetic nephropathy, while one trial failed to prove the effect [34]. There was moderate quality of evidence on improving UAER based on GRADE.

#### 3.3.2. Blood Urea Nitrogen

**Figure 2:**
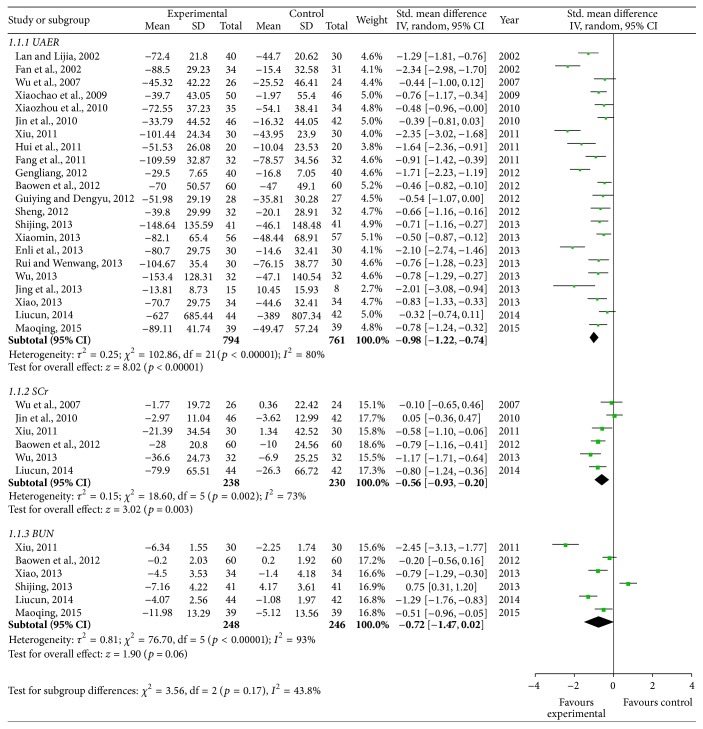
Forest plot showing the effect of Yiqi Yangyin Huoxue Method for diabetic nephropathy. UAER: urinary albumin excretion rate, SCr: serum creatinine, and BUN: blood urea nitrogen.

According to the blood urea nitrogen changes in forest plot, the effect size of the 6 selected trials [44, 49, 54, 55, 58, 59] was SMD = −0.72, 95% CI −1.47 to 0.02; test of the effect size was *z* = 1.90, *p* = 0.06; and the heterogeneity analysis (*p* < 0.05) suggests that there is heterogeneity in 6 articles. The aggregated results of 6 RCTs suggested that Yiqi Yangyin Huoxue Method did not show favorable effects for lowering blood urea nitrogen level of diabetic nephropathy. There was moderate quality of evidence on improving blood urea nitrogen based on GRADE (Figure 2).

#### 3.3.3. Serum Creatinine

According to the serum creatinine changes in forest plot, the effect size of the 6 selected trials [37, 40, 44, 49, 56, 58] was SMD = −0.56, 95% CI −0.93 to −0.20; test of the effect size was *z* = 3.02, *p* = 0.003; the heterogeneity analysis (*p* = 0.002) suggests that there is heterogeneity in 6 articles. The aggregated results of 4 RCTs [44, 49, 56, 58] suggested that Yiqi Yangyin Huoxue Method showed favorable effects for decreasing serum creatinine of diabetic nephropathy while the other 2 trials [37, 40] failed to show this effect. There was moderate quality of evidence on improving serum creatinine based on GRADE (Figure 2).

#### 3.3.4. Beta-2 Microglobulin

**Figure 3:**
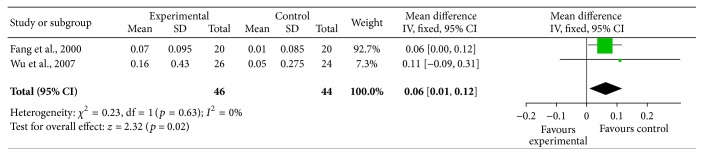
Forest plot showing the effect of Yiqi Yangyin Huoxue Method for diabetic nephropathy in beta-2 microglobulin.

According to the blood urea nitrogen changes in forest plot, the effect size of the 2 selected trials [36, 37] was MD = 0.06, 95% CI 0.01 to 0.12; test of the effect size was *z* = 2.32, *p* = 0.02; the heterogeneity analysis (*p* > 0.05) suggests that there is homogeneity in 2 articles. The aggregated results of 2 RCTs suggested that Yiqi Yangyin Huoxue Method showed favorable effects for bringing down beta-2 microglobulin of diabetic nephropathy. There was moderate quality of evidence on improving beta-2 microglobulin based on GRADE (Figure 3).

#### 3.3.5. Fasting Blood Glucose

**Figure 4:**
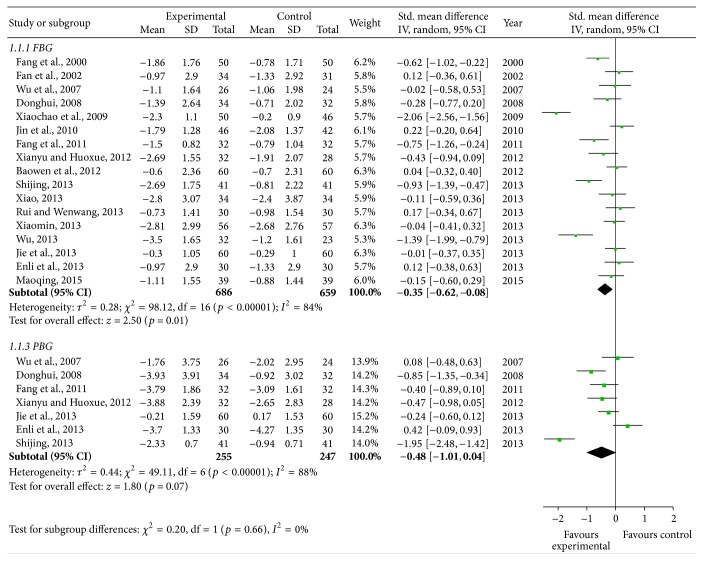
Forest plot showing the effect of Yiqi Yangyin Huoxue Method for diabetic nephropathy. FBG: fasting blood glucose; PBG: postprandial blood glucose.

According to the fasting blood glucose changes in forest plot, the effect size of the 17 selected trials [34, 36–40, 43, 45, 49–54, 56, 57, 59] was MD = −0.35, 95% CI −0.62 to −0.08; test of the effect size was *z* = 2.50, *p* = 0.01; the heterogeneity analysis (*p* < 0.05) suggests that there is heterogeneity in 17 articles. The aggregated results of 17 RCTs suggested that Yiqi Yangyin Huoxue Method showed favorable effects for decreasing fasting blood glucose of diabetic nephropathy. There was moderate quality of evidence on improving fasting blood glucose based on GRADE (Figure 4).

#### 3.3.6. Two-Hour Postprandial Blood Glucose

According to the 2-hour postprandial blood glucose (2hPBG) changes in forest plot, the effect size of the 7 selected trials [37, 38, 43, 45, 50, 53, 55] was SMD = −0.48, 95% CI −1.01 to 0.04; test of the effect size was *z* = 1.80, *p* = 0.07; the heterogeneity analysis (*p* < 0.05) suggests that there is heterogeneity in 7 articles. The aggregated results of 7 RCTs suggested that Yiqi Yangyin Huoxue Method did not show favorable effects for decreasing 2-hour postprandial blood glucose of diabetic nephropathy (Figure 4). There was moderate quality of evidence on improving 2-hour postprandial blood glucose based on GRADE.

### 3.4. Efficiency Analysis

In the end of the course of treatment, the aggregated results of 22 RCTs [35–37, 39–44, 46–52, 54–59] (SMD = −0.98, 95% CI −1.22 to −0.74) and test of the effect size (*z* = 8.02, *p* < 0.00001) suggested that Yiqi Yangyin Huoxue Method showed favorable effects for decreasing microalbuminuria of diabetic nephropathy; the aggregated results of 6 RCTs [44, 49, 54, 55, 58, 59] (SMD = −0.72, 95% CI −1.47 to 0.02) and test of the effect size (*z* = 1.90, *p* = 0.06) suggested that Yiqi Yangyin Huoxue Method did not show favorable effects for decreasing blood urea nitrogen; the aggregated results of 6 RCTs [37, 40, 44, 49, 56, 58] (SMD = −0.56, 95% CI −0.93 to −0.20) and test of the effect size (*z* = 3.02, *p* = 0.003) suggested that Yiqi Yangyin Huoxue Method showed favorable effects for decreasing serum creatinine; the aggregated results of 2 RCTs [36, 37] (MD = 0.06, 95% CI 0.01 to 0.12) and test of the effect size (*z* = 2.32, *p* = 0.02) suggested that Yiqi Yangyin Huoxue Method showed favorable effects for decreasing beta-2 microglobulin; the aggregated results of 17 RCTs [34, 36–40, 43, 45, 49–54, 56, 57, 59] (SMD = −0.35, 95% CI −0.62 to −0.08) and test of the effect size (*z* = 2.50, *p* = 0.01) suggested that Yiqi Yangyin Huoxue Method showed favorable effects for decreasing fasting blood glucose; and the aggregated results of 7 RCTs [37, 38, 43, 45, 50, 53, 55] (SMD = −0.48, 95% CI −1.01 to 0.04) and test of the effect size (*z* = 1.80, *p* = 0.07) suggested that Yiqi Yangyin Huoxue Method showed favorable effects for decreasing 2-hour postprandial blood glucose of diabetic nephropathy.

### 3.5. Heterogeneity Analysis

The heterogeneity analysis on the 22 trials of microalbuminuria suggests that there is heterogeneity (*p* < 0.00001, *p* < 0.05). The heterogeneity analysis on the 6 trials of blood urea nitrogen indicates that there is heterogeneity (*p* < 0.00001, *p* < 0.05). The heterogeneity analysis on the 6 trials of serum creatinine suggests that there is heterogeneity (*p* < 0.00001, *p* < 0.05). The heterogeneity analysis on the 2 trials of beta-2 microglobulin suggests that there is homogeneity (*p* = 0.63, *p* > 0.05). The heterogeneity analysis on the 17 trials of fasting blood glucose suggests that there is heterogeneity (*p* = 0.02, *p* < 0.05). The heterogeneity analysis on the 7 trials of 2-hour postprandial blood glucose suggests that there is heterogeneity (*p* = 0.02, *p* < 0.05).

## 4. Discussion

Current systematic review suggested that Yiqi Yangyin Huoxue Method showed positive effects for diabetic nephropathy on improving UAER, serum creatinine, fasting blood glucose, and beta-2 microglobulin, but not for blood urea nitrogen or PBG. However, there was heterogeneity in UAER, serum creatinine, fasting blood glucose, blood urea nitrogen, and PBG in the meta-analysis. Methodological quality of some studies was relatively low (only 2 points), but there was moderate quality of evidence on improving these outcomes based on GRADE.

Diabetic nephropathy (DN) is one of the most common chronic complications of diabetes. Severe microvascular pathological changes are common in diabetic patients with a history of more than 10 years. It is a major cause of death in patients with type 1 diabetes and its severity is second only to heart, cerebrovascular diseases in patients with type 2 diabetes. In TCM, the pathogenesis of diabetic nephropathy is deficiency of qi and yin and qi failing to circulate blood [60]. So the principle of treatment is to nourish qi and yin and promote blood circulation to resolve blood stasis. Drugs that function to tonify the kidney, nourish yin, reinforce spleen qi, and circulate blood (to resolve stasis) were often included in the formulas. The patients with edema were usually associated with drugs that function to resolve swelling. The outcome measures are UAER, urine beta-2 microglobulin, blood sugar, and so forth. In recent years, there are more and more randomized controlled trials on Yiqi Yangyin Huoxue Method for the treatment of diabetic nephropathy, which bring about opportunities for objective and comprehensive evaluation of the effect of this method, so this study conducted a meta-analysis to investigate its efficacy by the way of evidence-based medicine [61, 62].

This systematic review found that the present trials of Yiqi Yangyin Huoxue Method for the treatment of diabetic nephropathy were poor in methodology, blinding, allocation concealment, and randomization. In our study, all eligible trials included were Chinese. The course of the disease ranged from 18 months to 220.8 months and that of treatment from 3 to 12 weeks. 17 out of the 26 RCTs were high quality trials (3–5 points), and the rest were relatively low (only 2 points). Randomization was conducted in all articles, of which there are 2 with allocation concealment and 7 mentioning the blinding, and the withdrawals and dropouts or resign were mentioned in 4 trials. Though the quality of the included trials was relatively low, our review still provides stronger evidence of Yiqi Yangyin Huoxue Method for the treatment of diabetic nephropathy. Our systematic review showed the positive evidence that Yiqi Yangyin Huoxue Method had favorable effects for lowering the urine trace albumin excretion, serum creatinine, urine beta-2 microglobulin, fasting plasma glucose, and 2-hour postprandial blood glucose.

Our results are different from previous systematic reviews. Yan's [63] systematic review suggested that Yiqi Huoxue Qingre (invigorating qi and yin and activating blood) Method seemed to be an effective intervention in diabetic nephropathy. However, it did not meet the recognized pathogenesis for DN. The onset and process of DN are both deficiency of qi and yin and blood stasis, and Yiqi Huoxue Qingre therapeutic principle should be to tonify qi and yin and activate blood. In addition, the outcome measures were more considerate compared with Li's [64] review. Two-hour postprandial blood glucose was employed as an outcome because it is an important factor in the treatment of diabetic nephropathy. With more detailed subgroup analyses and comprehensive outcome measures, our update provides stronger evidence of Yiqi Yangyin Huoxue Method for diabetic nephropathy.

In our review, heterogeneity was found in the microalbuminuria excretion, serum creatinine, urine beta-2 microglobulin, fasting plasma glucose, and so forth of the included trials. The heterogeneity may be possibly from the risk of bias because effect estimate from studies that have low risk of bias might be different comparing to studies having high risk of bias. The reasons of the heterogeneity may be as follows: (1) the course of disease ranged from 18 to 220.8 months; (2) the course of treatment ranged from 3 to 12 weeks; (3) the outcome measures were different in the eligible trials; (4) the age and complications were different; and (5) the drugs used in Yiqi Yangyin Huoxue Method were also different. Firstly, antidiabetics and insulin were used in routine treatment of different trials. Secondly, the drugs used in the formula of Yiqi Yangyin Huoxue Method were different. Usually, drugs that were characterized with blood activating include* Salvia miltiorrhiza*, rhubarb, angelica, motherwort, red peony root, rhizoma ligustici wallichii, peach kernel, and cortex moutan; drugs that were characterized with invigorating qi include* Astragalus*, yam,* Atractylodes*, radix pseudostellariae, and dangshen; and drugs that were characterized with enriching yin include raw radix rehmanniae, cooked rehmannia, wolfberry fruit, radix scrophulariae, rhizoma polygonati, dogwood, and fructus ligustri lucidi. The drugs in each prescription therapeutic principle in the study of the trials were the same, but the type, dose, and origin were not identical.

Our study found that Yiqi Yangyin Huoxue Method had favorable effects for diabetic nephropathy; however, there is a major limitation for the included trials, that is, a small sample, and the quality of the trials was relatively low in methodology. Few studies observed the side effects of the treatment; thus the conclusions on the effect and safety should be interpreted with caution. More studies of Yiqi Yangyin Huoxue Method for diabetic nephropathy with large scale, high quality, and long follow-up are warrant to confirm the current findings.

## 5. Conclusion

Yiqi Yangyin Huoxue Method should be a valid complementary and alternative therapy in the management of diabetic nephropathy, especially on improving UAER, serum creatinine, fasting blood glucose, and beta-2 microglobulin. However, more studies with long follow-up are warrant to confirm the current findings.

## Figures and Tables

**Table 1:** The characteristics of the eligible studies.

Year	First author	Nation	Course of disease (month)	Case (T/C)	Intervention group	Control group	Course of treatment (week)	Outcome measures	Follow-up (month)
2000	Fang [34]	China	103.2	50/50	Yiqi Yangyin Huoxue + routine treatment	Routine treatment	8	Symptom score, FBG, UAER, and urine beta-2 microglobulin	NMT

2002	Lan [35]	China	60~120	40/30	Yiqi Yangyin Huoxue + routine treatment	Routine treatment + pancreatic kininogenase enteric-coated tablets	4	UAER, Symptom score	NMT

2002	Fan [36]	China	69.36 ± 24.08	34/31	Yiqi Yangyin Huoxue + routine treatment	Routine treatment	8	Symptom score, UAER, and FBG	NMT

2007	Wu [37]	China	NMT	26/24	Yiqi Yangyin Huoxue + routine treatment	Routine treatment	8	Symptoms, FBG, 2hPBG, and UAER	NMT

2008	Si [38]	China	220.8	34/32	Yiqi Yangyin Huoxue + routine treatment	Routine treatment	3~4	UAER, FBG, and PBG	NMT

2009	Xiaochao [39]	China	111.6	50/46	Yiqi Yangyin Huoxue + routine treatment	Routine treatment	8	Symptom score, UAER, and blood sugar	NMT

2010	Jin [40]	China	120	46/42	Yiqi Yangyin Huoxue + routine treatment	Routine treatment	8	Symptom score, UAER, FBG, and creatinine	NMT

2010	Xiaozhou [41]	China	158.4	35/34	Yiqi Yangyin Huoxue + routine treatment	Routine treatment	12	UAER, FBG, and creatinine	NMT

2011	Hui [42]	China	48~114	20/20	Yiqi Yangyin Huoxue + routine treatment	Routine treatment	4	UAER	NMT

2011	Fang [43]	China	134.4	32/32	Yiqi Yangyin Huoxue + routine treatment	Routine treatment	8	FBG, 2hPBG, and UAER	NMT

2011	Xiu [44]	China	122.16~171	30/30	Yiqi Yangyin Huoxue + routine treatment	Routine treatment	12	UAER, urea nitrogen, and creatinine	NMT

2012	Xianyu [45]	China	NMT	32/28	Yiqi Yangyin Huoxue + routine treatment	Routine treatment	8	FBG, 2hPBG, and UAER	NMT

2012	Sheng [46]	China	82.8~97.2	32/32	Yiqi Yangyin Huoxue + routine treatment	Routine treatment	8	UAER, urea nitrogen, creatinine, and FBG	NMT

2012	Guiying [47]	China	100.2	28/27	Yiqi Yangyin Huoxue + routine treatment	Routine treatment	8	Symptom score, UAER	NMT

2012	Gengliang [48]	China	72~94	40/40	Yiqi Yangyin Huoxue + routine treatment	Routine treatment	8	UAER	NMT

2012	Baowen [49]	China	18~96	60/60	Yiqi Yangyin Huoxue + routine treatment	Routine treatment	12	FBG, UAER, urea nitrogen, and creatinine	NMT

2013	Enli [50]	China	14.75~20.06	30/30	Yiqi Yangyin Huoxue + routine treatment	Routine treatment	8	FBG, 2hPBG, and UAER	NMT

2013	Xiaomin [51]	China	15.6~291.6	60/60	Yiqi Yangyin Huoxue + routine treatment	Routine treatment + benazepril	8	FBG, UAER	NMT

2013	Jing [52]	China	NMT	27/13	Yiqi Yangyin Huoxue + routine treatment	Routine treatment +placebo	12	UAER	NMT

2013	Jie [53]	China	68.4~108	60/60	Yiqi Yangyin Huoxue + routine treatment	Routine treatment + benazepril	8	FBG, 2hPBG	NMT

2013	Xiao [54]	China	59.04~136.68	34/34	Yiqi Yangyin Huoxue + routine treatment	Routine treatment	8	FBG, UAER, urea nitrogen, and creatinine	NMT

2013	Shijing [55]	China	8~180	41/41	Yiqi Yangyin Huoxue + routine treatment	Routine treatment	8	FBG, 2hPBG, UAER, urea nitrogen, and creatinine	NMT

2013	Wu [56]	China	36~204	32/32	Yiqi Yangyin Huoxue + routine treatment	Routine treatment	12	FBG, UAER, and creatinine	NMT

2013	Rui [57]	China	1~156	30/30	Yiqi Yangyin Huoxue + routine treatment	Routine treatment	12	FBG, UAER	NMT

2014	Liucun [58]	China	31.02~134.4	44/42	Yiqi Yangyin Huoxue + routine treatment	Routine treatment	8	UAER, urea nitrogen, and creatinine	NMT

2015	Maoqing [59]	China	12~180	39/39	Yiqi Yangyin Huoxue + routine treatment	Routine treatment	24	FBG, UAER, and urea nitrogen	2015

NMT: not mentioned; FBG: fasting blood-glucose; UAER: urinary albumin excretion rate; PBG: postmeal blood glucose.

**Table 2:** Methodological quality assessment.

Year	First author	Randomization	Blinding	Allocation concealment	Withdrawals and dropouts	Reason of dropouts and withdrawals	Jadad score
2000	Fang [34]	Yes	Yes	NMT	NMT	No	3
2002	Lan [35]	Yes	No	NMT	NMT	No	2
2002	Fan [36]	Yes	No	NMT	NMT	No	2
2007	Wu [37]	Yes	No	NMT	NMT	No	2
2008	Si [38]	Yes	No	NMT	NMT	No	2
2009	Xiaochao [39]	Yes	No	NMT	NMT	No	2
2010	Jin [40]	Yes	No	NMT	NMT	No	2
2010	Xiaozhou [41]	Yes	Yes	NMT	MT	Yes	4
2011	Hui [42]	Yes	No	NMT	NMT	No	2
2011	Fang [43]	Yes	Yes	NMT	NMT	No	3
2011	Xiu [44]	Yes	Yes	MT	NMT	No	4
2012	Xianyu [45]	Yes	No	NMT	NMT	No	2
2012	Sheng [46]	Yes	No	NMT	NMT	No	2
2012	Guiying [47]	Yes	Yes	NMT	MT	Yes	4
2012	Gengliang [48]	Yes	Yes	NMT	MT	Yes	4
2012	Baowen [49]	Yes	Yes	MT	MT	Yes	5
2013	Enli [50]	Yes	No	NMT	NMT	No	2
2013	Xiaomin [51]	Yes	No	NMT	NMT	No	2
2013	Jing [52]	Yes	No	NMT	NMT	No	2
2013	Jie [53]	Yes	No	NMT	NMT	No	2
2013	Xiao [54]	Yes	No	NMT	NMT	No	2
2013	Shijing [55]	Yes	No	NMT	NMT	No	2
2013	Wu [56]	Yes	No	NMT	NMT	No	2
2013	Wu [57]	Yes	No	NMT	NMT	No	2
2014	Liucun [58]	Yes	No	NMT	NMT	No	2
2015	Maoqing [59]	Yes	No	NMT	NMT	No	2

MT: mentioned; NMT: not mentioned.
